# Adaptive Text Messaging for Postpartum Risky Drinking: Conceptual Model and Protocol for an Ecological Momentary Assessment Study

**DOI:** 10.2196/36849

**Published:** 2022-04-04

**Authors:** Sarah Dauber, Alexa Beacham, Cori Hammond, Allison West, Johannes Thrul

**Affiliations:** 1 Partnership to End Addiction New York, NY United States; 2 Johns Hopkins Bloomberg School of Public Health Baltimore, MD United States; 3 Sidney Kimmel Comprehensive Cancer Center at Johns Hopkins Baltimore, MD United States; 4 Centre for Alcohol Policy Research La Trobe University Melbourne Australia

**Keywords:** postpartum, alcohol use, risky drinking, mobile health, ecologic momentary assessment, mobile phone

## Abstract

**Background:**

Risky drinking is prevalent among women of childbearing age. Although many women reduce their drinking during pregnancy, more than half return to prepregnancy levels during the early postpartum period. Risky drinking in new mothers may be associated with negative child and maternal health outcomes; however, new mothers are unlikely to seek treatment for risky drinking because of stigma and fear of child protective service involvement. SMS text messaging is a promising approach for reaching non–treatment-seeking new mothers at risk because of risky drinking. SMS text messaging interventions (TMIs) are empirically supported for alcohol use, but a tailored intervention for new mothers does not exist. This study aims to fill this gap by developing a just-in-time adaptive TMI for postpartum risky drinking.

**Objective:**

The objectives of this paper are to present a preliminary conceptual model of postpartum risky drinking and describe the protocol for conducting an ecological momentary assessment (EMA) study with new mothers to inform the refinement of the conceptual model and development of the TMI.

**Methods:**

This paper presents a preliminary conceptual model of postpartum risky drinking based on the motivational model of alcohol use, social cognitive theory, and temporal self-regulation theory. The model proposes three primary intervention targets: motivation, self-efficacy, and self-regulation. Theoretical and empirical literature in support of the conceptual model is described. The paper also describes procedures for a study that will collect EMA data from 30 participants recruited via social media and the perinatal Central Intake system of New Jersey. Following the baseline assessment, EMA surveys will be sent 5 times per day for 14 days. The assessment instruments and data analysis procedures are described.

**Results:**

Recruitment is scheduled to begin in January 2022 and is anticipated to conclude in March 2022. Study results are estimated to be published in July 2022.

**Conclusions:**

The study findings will enhance our understanding of daily and momentary fluctuations in risk and protective factors for risky drinking during the early postpartum period. The findings will be used to refine the conceptual model and inform the development of the TMI. The next steps for this work include the development of intervention components via an iterative participatory design process and testing of the resulting intervention in a pilot microrandomized trial.

**International Registered Report Identifier (IRRID):**

PRR1-10.2196/36849

## Introduction

### Background and Rationale

Risky drinking, defined as the consumption of ≥4 drinks in a day or ≥8 drinks in a week [1-3], is prevalent among women of childbearing age. A 2019 national survey found that, among women aged 18-25 years, 55% reported past-month alcohol use, 34% reported past-month binge drinking (defined as drinking ≥4 drinks at once), and 7% reported past-month heavy alcohol use (defined as drinking ≥4 drinks at once at least 5 days in the previous month). Among women aged 26-44 years, 59% reported past-month alcohol use, 30% reported past-month binge drinking, and 6% reported past-month heavy alcohol use [4]. Although many women reduce their drinking during pregnancy, more than half return to prepregnancy levels by 3 months after delivery [5,6]. Patterns of postpartum drinking vary widely, with 8%-12% of women showing patterns of escalating risky drinking in the early postpartum period [7-9]. Depending on severity, postpartum alcohol use can lead to impaired parenting and an increased risk of child maltreatment [10], which can have devastating impacts on brain development, leading to long-term impairment [11,12]. Given the increase in drinking and the potential for severe consequences to the child [13-15], the postpartum period is a critical time for intervention to address risky drinking. However, most adults who engage in risky drinking do not seek treatment [16]. New mothers may be particularly unlikely to seek treatment because of stigma and fear of child protective service involvement [17]. Thus, there is a critical need for innovative approaches to reach non–treatment-seeking new mothers at high risk for negative consequences associated with risky drinking. The goal of this study is to meet this need by developing an SMS text messaging intervention (TMI) to address postpartum risky drinking. In this paper, we describe two critical first steps toward this goal: (1) development of a preliminary conceptual model of postpartum risky drinking and (2) presentation of a protocol for data collection via ecological momentary assessment (EMA) to refine the conceptual model and inform TMI development.

SMS text messaging is a promising approach for reaching non–treatment-seeking risky drinkers and may be particularly suitable for addressing postpartum risky drinking. With 97% of Americans owning a cell phone as of 2021, access to SMS text messaging is widespread [18]. Studies suggest that 99% of received SMS text messages are opened, and 90% are read within 3 minutes of receipt [19]. TMIs are highly acceptable to people with drug and alcohol dependence [20] and have high scalability potential at a relatively low cost. Of particular importance for new mothers, TMIs provide a way of delivering interventions anonymously, potentially overcoming the stigma and fear of consequences that often prevent mothers from accessing more traditional forms of treatment [17,21-24]. In a recent survey, low-income new mothers with histories of risky drinking reported high levels of mobile phone ownership and use of SMS text messaging as well as favorable reactions to receiving SMS text messages to address alcohol use [25].

A growing body of literature demonstrates support for TMIs in reducing risky drinking in non–treatment-seeking adults [20,26-30]. In a recent review of mobile health interventions for unhealthy alcohol use, over half of the TMIs reviewed were effective in reducing alcohol use or increasing readiness to change [31]. However, the existing literature on TMIs for alcohol use has several limitations, including a lack of theoretical behavior change models guiding intervention design, small samples, and lack of long-term follow-up [20,21,32]. In addition, most existing studies have compared whole TMIs to attention- or assessment-only controls and were not able to disentangle the impacts of individual intervention components. Generalizability is also limited as most studies have focused on college drinkers, adults with alcohol use disorder, or patients in the emergency department. There are currently no TMIs for alcohol use that are tailored to the unique characteristics of the postpartum period.

TMIs that tailor content to specific participant characteristics or clinical needs show larger effects on clinical outcomes and lower rates of attrition than programs that deliver generic messages [20,26,33]. Interventions that are dynamically tailored in response to reports of changing needs or other variables assessed regularly throughout intervention delivery are referred to as *just-in-time adaptive interventions* (JITAIs). JITAIs aim to provide interventions at times when individuals are particularly vulnerable and when opportunity for positive change is greatest [34], making this approach a good fit for the postpartum period. The postpartum period is inherently a time of high vulnerability, as demonstrated by higher rates of depression and perceived stress among new mothers [35], which are known risk factors for alcohol use [36-38]. New mothers experience unique transient influences on vulnerability, including daily stress associated with caring for a new baby that can be exacerbated by factors such as lack of sleep and baby irritability [39-41]. The postpartum period is also a time of opportunity for positive change. Pregnant and postpartum people generally report high levels of motivation to change behaviors that may negatively affect their baby, making this time of life a *teachable moment* with maximum potential for effecting positive behavior change [42].

This study will apply the multiphase optimization strategy (MOST) framework [43] to develop and pilot-test the first theory-driven just-in-time adaptive TMI for postpartum risky drinking. The MOST is an engineering-inspired framework that allows for the identification of the optimal package of intervention components and is recommended for building efficient, scalable mobile health interventions [44]. The MOST proceeds in 3 phases. The preparation phase, which is the focus of this study, is aimed at (1) developing a conceptual model that specifies the relationships between intervention components, intervention targets, and outcomes; (2) developing content and delivery strategies for each intervention component; and (3) pilot-testing the developed intervention components. In the optimization phase, the intervention components are further tested in an efficient experimental design with the goal of identifying the best combination of components [45]. Finally, the evaluation phase consists of a traditional randomized controlled trial comparing the full intervention package with a suitable control group.

Following the MOST framework, the first step in developing a theory-driven JITAI for postpartum risky drinking is to specify the theoretical pathways from the postpartum risk factors that are the primary intervention targets to the ultimate desired outcome of reduced risky drinking. Our proposed conceptual model (described in the *Methods* section) is based on three theoretical frameworks that have been widely used to explain alcohol and substance use: the motivational model of alcohol use [46], social cognitive theory [47], and temporal self-regulation theory [48]. These theories have been used previously in the development of TMIs for alcohol use [49-52] but have never been applied to risky drinking in the postpartum period. On the basis of these theories, we have identified three core intervention targets: motivation, defined as commitment to avoid drinking [53]; self-efficacy, including self-efficacy to avoid drinking and self-efficacy in the maternal role; and self-regulation, defined by the use of a range of adaptive coping strategies. All 3 intervention targets correspond to behavior change techniques that have demonstrated efficacy in the context of brief interventions for alcohol use [54-56]. All 3 theoretical frameworks describe internal and external contextual variables operating as risk and protective factors for the intervention targets that ultimately affect drinking behavior. On the basis of the theoretical frameworks as well as the limited empirical literature on the postpartum period, we have selected the following internal and external factors for inclusion: mood, stress, and fatigue (internal factors), and baby fussiness, social support, and drinking cues (external factors). Empirical literature supporting the conceptual model and the selection of contextual variables is described in the *Methods* section.

As JITAIs aim to intervene at the momentary level, a comprehensive understanding of the daily and momentary fluctuations in risks and protective factors for postpartum risky drinking is needed to inform the design of a tailored JITAI for this population. EMAs collect data in real time over the course of a day and are designed to capture momentary fluctuations in feelings and behaviors as participants go about their daily lives [57]. EMAs are particularly suitable for tracking changes in state-level characteristics, which are thought to change significantly within short periods. This method has been widely used in research on substance use and other health behaviors [57-59]. In total, 2 EMA studies with new mothers [60,61] offer preliminary support for the feasibility of this approach with this population. However, almost nothing is known about the in-the-moment predictors of daily drinking in the postpartum period, information that is crucial for the design of effective interventions for this population.

### Objectives

The objectives of this paper are to (1) present a preliminary conceptual model of postpartum risky drinking and (2) describe the protocol for conducting an EMA study with new mothers to inform the refinement of the conceptual model and the development of a just-in-time adaptive TMI to address postpartum risky drinking.

The purpose of the EMA study is to assess and refine the conceptual model and test the feasibility of EMA data collection procedures in a sample of new mothers. The primary research questions of the EMA study are as follows: (1) How do momentary and daily fluctuations in internal and external contextual factors affect motivation, self-efficacy, and self-regulation? (2) Which internal and external factors are most salient at particular times of the day? (3) What is the relationship between maternal self-efficacy and drinking self-efficacy and how does this relationship fluctuate throughout the day? (4) What is the relationship between momentary and daily changes in motivation, self-efficacy, and self-regulation and daily drinking? (5) To what extent are the study methods (eg, number and length of surveys and item wording) acceptable and feasible for the target population of new mothers within the early postpartum period?

## Methods

### Project Overview

**Figure 1:**
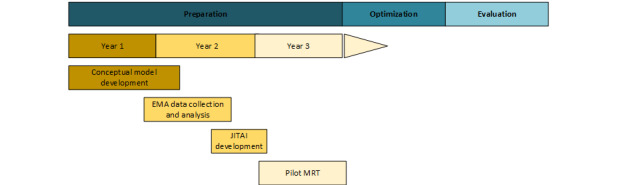
Study timeline. EMA: ecological momentary assessment; JITAI: just-in-time adaptive intervention; MRT: microrandomized trial.

This EMA study is part of a 3-year effort to develop a JITAI for postpartum risky drinking that comprises the preparation phase of the MOST framework (Figure 1). EMA data collection represents the first stage of this work, aimed at refining a conceptual model of postpartum risky drinking and informing JITAI development. This paper presents our preliminary conceptual model of postpartum risky drinking and our protocol for EMA data collection. Following completion of the EMA, components of the JITAI will be developed via an iterative participatory design process with focus groups of new mothers. Finally, the resulting JITAI will be tested in a pilot microrandomized trial.

### Ethics Approval

The study was approved by the Solutions Institutional Review Board in October 2020 (#2020/06/15) and registered at ClinicalTrials.gov. All study participants will provide informed consent to take part.

### Conceptual Model

#### Overview

**Figure 2:**
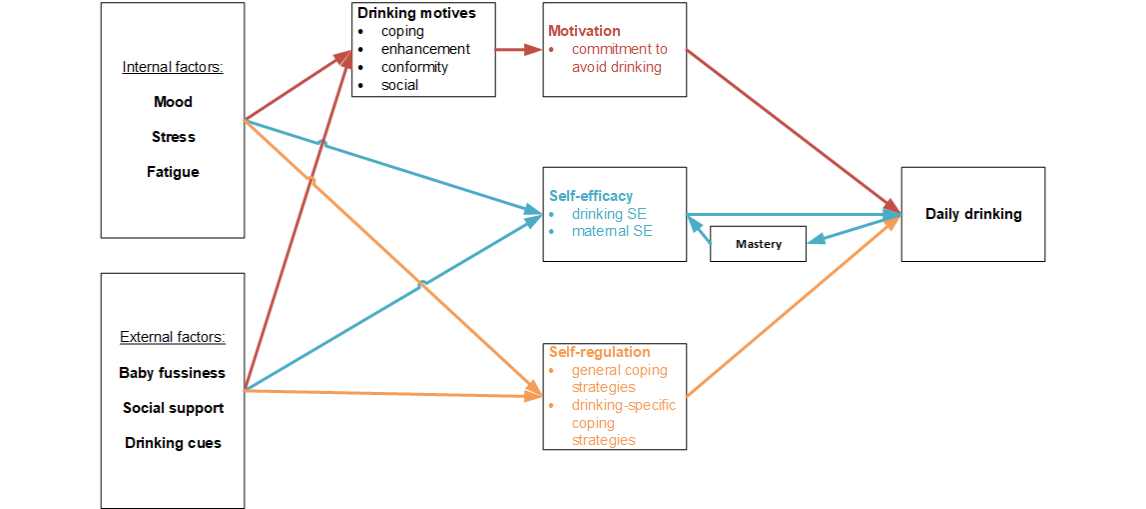
Preliminary conceptual model of postpartum risky drinking. SE: self-efficacy.

Our preliminary conceptual model was developed via a review of relevant theoretical and empirical literature combined with a series of brainstorming conversations among the study team. Figure 2 depicts the proposed conceptual model of postpartum risky drinking that guided the development of our EMA data collection protocol. This model is preliminary and subject to adjustment based on the findings of the EMA study. Drawing from the motivational model of alcohol use [46], social cognitive theory [47], and temporal self-regulation theory [48], this model identifies three core intervention targets (motivation, self-efficacy, and self-regulation) that have been reliably assessed at the momentary or daily level in EMA studies. This section describes the empirical literature that supports our conceptual model organized according to the intervention target.

#### Motivation

According to the motivational model of alcohol use, motivation to drink is the most proximal predictor of drinking behavior [46]. Our conceptual model operationalizes motivation as commitment to avoid drinking, consistent with other studies examining within-day fluctuations in motivation as a key mechanism of change in substance use treatment [53,62,63]. Measured in this way, motivation has been found to fluctuate within a single day, and these changes were associated with drinking the following day [53].

The motivational model proposes four types of drinking motives that may affect within-day fluctuations in motivation to drink: coping (aimed at reducing negative emotions), enhancement (aimed at increasing positive emotions), conformity (aimed at avoiding social rejection), and social (aimed at increasing positive social experiences), with varying antecedents and consequences of each [64]. Findings from EMA studies of alcohol motivations demonstrate that motives vary within persons and across time and situations in response to internal and external contextual factors [65]. Previous EMA studies suggest that higher positive affect is associated with greater enhancement motives [66-68] and higher negative affect is associated with greater coping motives at the daily level [69,70]. Studies examining links between drinking motives and outcomes have shown that daily enhancement motives are generally associated with poorer daily drinking outcomes [68,71,72]. Findings for coping motives are less consistent, with some studies finding that coping motives are associated with increased quantity and severity of alcohol use [71,72] and others finding no relationship [67,68,73]. Most existing research has been conducted with college student samples, and there may be different patterns among new mothers, which we will begin to elucidate in this study.

#### Self-efficacy

Consistent with social cognitive theory, the conceptual model suggests that two types of self-efficacy—drinking self-efficacy and maternal self-efficacy—may contribute to drinking behavior. Drinking self-efficacy, defined as a person’s belief in their ability to avoid drinking, is well-supported as a significant predictor of drinking behavior. Higher drinking self-efficacy has been shown to predict less drinking and improved long-term outcomes in the context of treatment for alcohol use disorder [74-78]. Studies with individuals who engage in problematic drinking and are treatment-seeking have found that daily within-person change in self-efficacy to avoid drinking is associated with intensity of drinking the next day [53,62].

In addition to drinking self-efficacy, our conceptual model includes self-efficacy specific to the maternal role, defined as a mother’s belief in her ability to successfully care for her baby. There is currently no research examining associations between maternal self-efficacy and alcohol use. In addition, no study to date has examined daily or momentary changes in maternal self-efficacy during the postpartum period despite evidence that self-efficacy in other domains changes over brief periods [59,79,80]. This study will explore the associations between maternal self-efficacy and drinking at the daily and momentary levels to inform whether maternal self-efficacy may be an important intervention target for new mothers who engage in risky drinking.

Both drinking self-efficacy and maternal self-efficacy are influenced by internal and external contextual factors, as reflected in the conceptual model. Variations in mood, stress, and fatigue have been shown to affect both drinking self-efficacy [62,81,82] and maternal self-efficacy [83-85]. In addition, maternal self-efficacy is affected by difficult infant behavior [39,40,86,87] and a lack of social support [88-90]. The model also includes a feedback loop between self-efficacy, daily drinking, and mastery such that mastery experiences can increase self-efficacy, thereby reducing the likelihood of next-day drinking. For example, a successful attempt to avoid drinking is likely to increase an individual’s belief in their own capacity to avoid drinking in the future, and this heightened self-efficacy makes them more likely to succeed in future attempts to avoid drinking. Empirical studies on the mastery feedback loop are limited, but there is some support for the reciprocal relationship between mastery and self-efficacy in a sample of adults who smoke [91].

#### Self-regulation

According to temporal self-regulation theory, self-regulation, or the ability to monitor and adapt cognitions, emotions, and behaviors in response to internal or external contextual factors in a goal-directed manner, is a key factor affecting risky behaviors, including alcohol use [92,93]. Internal and external factors can act as triggers for substance use, and adaptive self-regulation strategies must be applied to avoid drinking alcohol in the presence of these triggers [94]. In addition, internal and external factors can predict the likelihood that a person will apply self-regulation strategies in a particular situation [93,95]. Self-regulation is a core theoretical mechanism of behavior change in cognitive behavioral treatments for addiction [96,97], although findings related to the effectiveness of specific self-regulatory strategies for alcohol use have been mixed [95].

A small number of studies have examined daily within-person changes in self-regulation strategies in the context of alcohol use [94]. Studies typically define self-regulation as the use of adaptive coping strategies [69,98,99] or protective drinking strategies [100,101]. Daily engagement in coping strategies has been associated with drinking behavior, with some studies finding differences based on the specific strategy used [69,98] and others not [102]. Given that nearly all studies have used college student samples and there is no research to guide the selection of specific strategies for new mothers, our study includes a broad range of self-regulation strategies with the aim of determining those most salient for our target population.

### Target Population, Eligibility Criteria, and Sample Size

The study target population is adults aged 18-45 years who live in New Jersey and gave birth to a live infant within the previous 2 weeks who is currently in their care. This study is being conducted in New Jersey to leverage existing partnerships between the study team and the New Jersey state system of care for perinatal women. Additional eligibility criteria include speaking English and access to a smartphone with internet. Participants must also report one of the following: (1) a score of ≥2 on the Tolerance, Annoyance, Cut Down, Eye-Opener (T-ACE) alcohol risk screener, (2) having ≥8 standard drinks in 1 week in the 12 months before becoming pregnant, or (3) having ≥4 drinks at one time once a month or more often in the 12 months before becoming pregnant. We aim to recruit 30 participants who meet the eligibility criteria. Similar sample sizes have been used in other EMA studies of individuals who engage in substance use [103-105] and new mothers [106].

### Recruitment, Eligibility Screening, and Informed Consent Procedures

This study will use two primary recruitment strategies: (1) recruitment via social media advertisements on Facebook and Instagram and (2) referrals from providers in the New Jersey perinatal Central Intake (CI) system.

#### Social Media Recruitment

Advertisements for the study will be placed on Facebook and Instagram and will be geographically targeted to New Jersey. Additional advertisement targeting will include interests related to birth, pregnancy, motherhood, infant care, and drinking alcohol. Individuals who click on an advertisement will be directed to the study website. Social media recruitment via Facebook and Instagram is widely used in research study recruitment and has been used successfully with both new mothers [107-109] and individuals who are using substances [110,111]. Individuals who access the study website via a social media advertisement will have the option to complete eligibility screening and informed consent on the web or to connect directly with the study coordinator and complete the process via phone.

#### CI Recruitment

New Jersey operates a state-wide CI system that provides a single point of entry into services for pregnant and postpartum people to promote improved care coordination and access to needed services. For this study, we will partner with one of the CI sites that serves a large, demographically diverse county in the state. CI workers will introduce the study to their clients using a script provided by the study team. If a client is interested in learning more about the study, the CI worker will provide their contact information to the study coordinator, who will contact the client within 2 weeks of her due date to complete eligibility screening. For clients who are interested in the study but prefer not to share their contact information, the CI worker will direct them to the study website, where they can complete eligibility screening and informed consent on the web.

### Baseline Survey and EMA Training

Eligible participants who complete the informed consent process will be invited to complete the baseline survey. Baseline survey data will be used to describe the study sample and understand the impact of baseline characteristics on momentary changes in the variables of interest. The baseline survey can be completed either on the web via Qualtrics (Qualtrics International Inc) or via phone with the study coordinator, depending on the participant’s preference. The baseline survey will take approximately 30 minutes to complete, and participants will receive a US $25 gift card upon completion. The baseline survey will assess demographic characteristics, maternal self-efficacy, mental health, stress and coping, motivation, alcohol use, and other substance use. See Table 1 for a complete list of the baseline measures. Following completion of the baseline survey, participants will complete a one-on-one 30-minute EMA training session with the study coordinator via Zoom. During the training, the study coordinator will instruct the participants in the installation and use of the MetricWire (MetricWire Inc) data collection app as well as in best practices for maintaining privacy throughout the study. Participants will be considered enrolled in the study after they complete both the baseline survey and EMA training.

**Table 1:** Baseline measures.

Construct	Description	Measure (reference)
Demographics	Age, gender, marital status, race, ethnicity, living arrangements, childbirth history, education, employment, income, and substance use treatment history	—^a^
Drinking self-efficacy	Perceived ability to handle various drinking situations	Drinking Refusal Self-efficacy Questionnaire–Revised [112]
Alcohol and drug use history	Use of alcohol, marijuana, and illegal drugs before pregnancy, during pregnancy, and since giving birth	Adapted from the NIDA^b^-modified ASSIST^c^ [113]; NIAAA^d^ drinking questions [114]
Motivation	Readiness to change alcohol use	Maternal Motivation Scale [115]
Postpartum depression	Symptoms of depression since giving birth	Beck Depression Scale [116]
Maternal self-efficacy	Confidence in carrying out various baby care tasks	Karitane Parenting Confidence Scale [117]
Trauma history	Experiences of trauma during childhood	Adverse Childhood Experiences Questionnaire [118]
Pandemic stress	Stress related to the COVID-19 pandemic	Adapted from the Pandemic Stress Index [119]
Attachment to infant	Mother experience of bonding and attachment to baby	Infant Bonding Scale [120]
Fatigue	Experiences of emotional and physical fatigue	Fatigue Assessment Scale [121]
Stress	Perceptions of stress related to general life experiences	Perceived Stress Scale–4 [122]
Drinking motives	Motivation to consume alcohol	Drinking Motives Questionnaire–Revised [64]
Coping self-efficacy	Perceived ability to cope with challenging life events	Coping Self-efficacy Scale [123]
Social support	Perceptions of social support	Interpersonal Support Evaluation List–12 [124]
Digital literacy	Comfort using technology to complete tasks, such as SMS text messaging, using a smartphone, and accessing health information on the web	Media and Technology Usage and Attitudes Scale [125]; the eHealth Literacy Scale [126]

^a^There is no specific citation for the demographic items.

^b^NIDA: National Institute on Drug Abuse.

^c^ASSIST: Alcohol, Smoking, and Substance Involvement Screening Test.

^d^NIAAA: National Institute on Alcohol Abuse and Alcoholism.

### EMA Data Collection Procedures

All EMA data will be collected via the MetricWire app. The MetricWire app is available for free download from the Apple App Store and Google Play Store and has been used in other EMA research studies [127,128]. The participants will use their own smartphones to complete the EMA surveys 5 times daily for 14 days. The five surveys will include a *morning survey*, which is a daily diary asking about the previous day, and 4 shorter *hourly surveys*, which ask questions about the period since the previous survey. The selection of 5 daily surveys is based on the need to balance desire to assess fluctuations in risk factors with considerations of participant burden [129], and 3-4 surveys a day has been found to be acceptable to young mothers [130].

**Figure 3:**
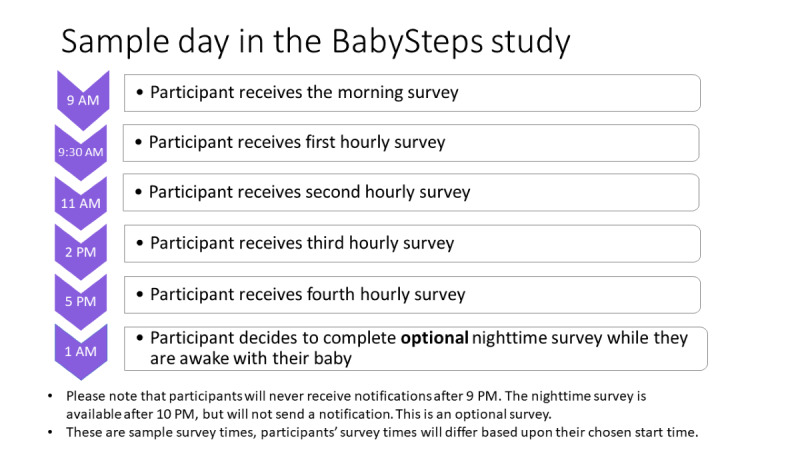
Sample schedule of daily survey prompts.

Figure 3 displays a sample daily schedule of EMA prompts. Upon enrollment in the study, the participants will be asked to select a morning start time for receiving messages each day. Each day, the morning survey will be sent within 1 hour after the selected start time and will ask questions about the previous day. The morning survey should take approximately 2-3 minutes to complete and will remain available for 10 hours before expiring.

The remainder of the day will be divided into 4 equal segments, and hourly surveys will be sent randomly within each segment. No surveys will be sent later than 9 PM. After each survey prompt, the survey will be available for up to 60 minutes, with 2 reminder prompts sent at 20 and 40 minutes. Surveys that are not completed within the 60-minute window will expire. Completion of each hourly survey will take 1-2 minutes.

Between the last survey in the evening and the first survey in the morning, an optional EMA survey will be available for the participants to complete. The reason for this optional survey is to enable data collection during the night, when the participants may be awake with their baby. Nights may be times of high stress and high risk of drinking for new mothers. This survey will allow us to capture data on these middle-of-the-night times without disturbing the participants by sending prompts. The participants who complete the night survey will receive an automatic response SMS text message with contact information for a 24-hour support hotline.

### Participant Remuneration

Study participants will be remunerated for taking part in the study in the form of gift cards to Amazon or Target. The participants will be paid US $25 for completion of the baseline survey. During EMA data collection, the participants will be paid US $2 for each EMA survey completed, with a bonus of US $20 for completing >50% of the EMA surveys and US $30 for completing >80% of the EMA surveys. Bonus incentives are used routinely in EMA studies to boost compliance and have been used in EMA studies with postpartum women [131]. The participants will be able to view their progress toward earning bonus incentives within the MetricWire app.

### Participant Support

To ensure that the participants are adequately supported during the study, we will engage in the following: (1) check in briefly by phone with all participants after 3 days of EMA to obtain initial feedback on the questions and address any technical difficulties, (2) provide information on how to obtain immediate support via hotlines, and (3) provide all participants with a list of local mental health and substance use treatment and support resources at the outset of the study. Information about how to obtain immediate support will be available within the MetricWire app at all times for the participants to access as needed.

### EMA Measures

The study team reviewed the existing literature and selected EMA measures that align with each construct in the conceptual model (Figure 1). As EMA measures must be brief, we prioritized measures that have been used in other EMA studies, particularly those used with a similar population. For measures that have not been used in previous EMA studies, we reviewed factor analyses of full-length scales and selected the highest-loading items to represent the constructs of interest. For some items, we adapted the wording to fit the momentary nature of the EMA. The final list of items included in the morning and hourly EMA surveys is shown in Table 2 organized according to the constructs in the conceptual model. At the beginning of each survey, the participant is asked to indicate how much time they spent with their baby the previous day (morning survey), whether they were with their baby since the previous survey, and whether they will be with their baby in the next hour (hourly survey). Responses determine which survey questions are asked based on skip patterns. For example, participants who report that they were not with their baby since the previous survey will not be asked how many times their baby fussed or cried since the last survey. The morning survey includes a total of 23 items, and the hourly surveys include a total of 14 items.

**Table 2:** Morning and hourly ecological momentary assessment (EMA) survey items and response options.

Construct, subcategories, and measure (reference)				Item (response options)		Morning		Hourly
**Internal factors**								
	**Mood**							
		Adapted from the studies by Nguyen et al [58], Godell et al [111], and Thrul et al [132]	What is your overall feeling right now? (1=very unpleasant to 7=very pleasant) What is your overall energy level right now? (1=very low to 7=very high) What is your anxiety level right now? (1=very low to 7=very high)				✓	
	**Stress**							
		Adapted from the Perceived Stress Scale [122]; single item used in previous EMA studies [58,61]	What is your overall stress level right now? (1=very low to 7=very high)				✓	
	**Fatigue**							
		Adapted from the Fatigue Assessment Scale [121]	How physically exhausted are you right now? (1=not at all to 7=extremely) How mentally exhausted are you right now? (1=not at all to 7=extremely)				✓	
		Adapted from the studies by Dennis and Ross [40] and Mendez et al [61]	How well did your baby sleep last night? (1=poor to 7=excellent) How well did you sleep last night? (1=poor to 7=excellent)		✓			
**External factors**								
	**Baby fussiness**							
		Selected items from the Infant Characteristics Questionnaire [133]	Yesterday, how easy or difficult was it for you to calm or soothe your baby when they were upset? (1=very easy to 7=very difficult) Yesterday, how much did your baby cry and fuss in general? (1=very little to 7=a lot)		✓			
		Adapted from the study by Adams et al [134]	Since the last survey, how many times did your baby fuss, cry, or seem upset? (0, 1, 2, 3, 4, 5, 6, 7, 8, 9, or 10 or more times) How many of these times were you able to successfully soothe your baby? (All of them; most of them; some of them; a few of them; none of them)				✓	
	**Social support**							
		Item from the Maternal Social Support Index [135]	How often was support available to you when you needed it yesterday? (None of the time; little of the time; some of the time; most of the time)		✓			
	**Drinking cues**							
		Adapted from the study by McQuoid et al [103]	Was alcohol available to you yesterday? (Yes or no) Were other people in your household drinking alcohol yesterday? (Yes or no)		✓			
**Motivation**								
	**Drinking motives**							
		Selected items from the Drinking Motives Questionnaire [64]	If you drank alcohol yesterday, why did you drink? (Because it makes social gatherings more fun; to forget about your problems; because it gives you a pleasant feeling; to be liked)		✓			
	**Commitment to avoid drinking**							
		Adapted from the study by Kuerbis et al [53]	How committed are you to not drink alcohol in the next hour (for hourly survey) or today (for morning survey)? (1=not at all to 7=extremely)		✓		✓	
**Self-efficacy**								
	**Maternal self-efficacy**							
		Developed for this study	How confident are you that you will be able to meet your baby’s physical needs (such as needs to be fed or changed) over the next hour (for hourly survey) or today (for morning survey)? (1=not at all confident to 7=extremely confident) How confident are you that you will be able to meet your baby’s emotional needs (such as needs to be soothed or entertained) over the next hour (for hourly survey) or today (for morning survey)? (1=not at all confident to 7=extremely confident)		✓		✓	
	**Drinking self-efficacy**							
		Adapted from the study by Kuerbis et al [53]	How confident are you that you can avoid drinking alcohol for the next hour (for hourly survey) or today (for morning survey)? (1=not at all confident to 7=extremely confident)		✓		✓	
**Self-regulation**								
	**Adaptive coping—general**							
		Adapted from the studies by Roos et al [94] and Cambron et al [136]	Did you use any strategies to cope with negative feelings or stress since the last survey (for hourly survey) or yesterday (for morning survey)? (I didn’t experience negative feelings or stress; I drank alcohol; I changed my thinking; I changed my current situation; I found something else to do; I sought advice or support; I came up with a plan to cope; I set a goal or kept track of my current progress toward a goal; I directly communicated my needs to others; I tried to relax; I took medication; I pushed negative feelings or stress away; I used another strategy; I didn’t use any strategies)		✓		✓	
	**Adaptive coping—drinking**							
		Adapted from the studies by Roos et al [94] and Cambron et al [136]	Did you use any strategies to manage the urge to drink alcohol since the last survey (for hourly survey) or yesterday (for morning survey)? (I didn’t experience an urge to drink alcohol; I changed my thinking; I changed my current situation; I found something else to do; I sought advice or support; I came up with a plan to manage the urge to drink alcohol; I set a goal or kept track of my current progress toward a goal; I directly communicated my needs to others; I tried to relax; I made an effort to stay safe and avoid risks while drinking alcohol; I took medication; I tried to ignore the urge to drink alcohol; I used another strategy; I didn’t use any strategies)					
**Mastery**								
	**Mastery—parenting**							
		Selected items from the Perceived Maternal Parenting Self-efficacy Tool [137]	Yesterday, I was good at feeding my baby (1=strongly disagree, 2=disagree, 3=agree, 4=strongly agree) Yesterday, I was good at soothing my baby when they became upset (1=strongly disagree, 2=disagree, 3=agree, 4=strongly agree) Yesterday, I was good at reading my baby’s cues (1=strongly disagree, 2=disagree, 3=agree, 4=strongly agree) Yesterday, my baby responded well to me (1=strongly disagree, 2=disagree, 3=agree, 4=strongly agree)		✓			
	**Mastery—drinking**							
		Developed for this study	How strong was your urge to drink alcohol yesterday? (1=very low to 7=very high) To what extent did you feel that you overcame your urge to drink alcohol yesterday? (1=not at all to 7=to a very great extent)		✓			
**Daily drinking**								
	**Drinking**							
		Adapted from the studies by O’Donnell et al [73] and Thrul et al [138]	Did you consume any alcohol yesterday? (Yes or no) If yes, how many alcoholic drinks did you consume? (Enter numeric value) If yes, select all the time periods when you drank alcohol yesterday (Morning; lunchtime; afternoon; evening; during the night)		✓			

### Acceptability and Feasibility

Drawing from other EMA feasibility studies [58,128,139-142], feasibility outcomes will include the percentage of EMA surveys completed each day, each week, and at the end of the 14-day period; the percentage of each EMA measure completed; and the percentage of respondents who completed 50% and 80% of the EMA surveys [58,139]. Acceptability measures will be collected via a short text-based survey at the end of the 14-day EMA period and will include an assessment of technical challenges, burden, emotional response, and overall satisfaction using Likert scale items from other EMA studies [128,140-142].

### Compliance Monitoring Strategies

We will apply the following established methods to encourage and monitor compliance: (1) one-on-one training on EMA procedures before the start of data collection [128,131,140,143-146], (2) availability of technical support by phone throughout the EMA period [131,140,144], (3) daily SMS text message reminders to complete the EMA surveys [143,144], (4) bonus incentives for completing >50% and >80% of the EMA surveys [131,143,144], (5) keeping the participants informed of their progress toward earning bonus incentives [143,144], and (6) outreach to participants who did not complete any EMA surveys 3 days in a row [144,146]. If 3 days pass without any EMA responses, the participants will receive an automated SMS text message reminding them to complete the surveys. The reminder message will be sent once per day for 5 days. If there is still no response, the participant will be considered dropped from the study.

### Data Analysis

As the primary purpose of the EMA is to inform the development of the JITAI, the analyses will be largely descriptive. To avoid issues of data quality stemming from noncompliance, we will exclude participants who complete <50% of the required EMA surveys [58]. On the basis of rates of EMA compliance in studies of young adults who drink alcohol [147,148] and of postpartum women [106,131], we expect that nearly all participants will complete ≥50% of the EMAs. We will use descriptive statistics to describe the baseline characteristics of the study sample as well as the feasibility outcomes. Patterns of missing data in the EMA surveys will be studied using frequency distributions and graphs to discern whether there are certain times when participants are more or less responsive to prompts. We will also examine the average time to respond following each prompt. As in other EMA studies [131], the variation in response to each variable will be graphed using scatter plots with a Loess smoother and examined visually to detect patterns in fluctuations within and across days. To test associations among variables, we will apply generalized estimating equations following the procedures used by Nguyen et al [58] and Thrul et al [132], testing linear and quadratic effects. Generalized estimating equations account for the nesting of multiple observations within participants [149]. To inform the selection of decision rules for the JITAI, we will examine associations among predictors (internal and external factors), mediators (intervention targets), and outcomes (daily drinking) based on individual EMA surveys and averaged across each day. To inform the selection of tailoring variables, we will examine variability in internal and external factors across EMA surveys as well as variability in their relations to the primary intervention targets (motivation, self-efficacy, and self-regulation). For these analyses, the internal and external factors will be examined as time-varying predictors of intervention targets as recommended by Shiffman [150]. We will assess participant differences in EMA feasibility and acceptability measures using independent sample 2-tailed *t* tests and repeated-measures analyses of variance.

## Results

Recruitment for this study is scheduled to begin in January 2022. We anticipate completing recruitment and enrollment by March 2022 and expect to have completed EMA data collection by April 1, 2022. Study results will be published in peer-reviewed scientific journals upon completion of data analysis, which is estimated to be in July 2022.

## Discussion

### Principal Findings

This paper presents a preliminary conceptual model of postpartum risky drinking as well as a protocol for an EMA data collection study aimed at refining the conceptual model and informing the development of the first JITAI for postpartum risky drinking. This study is the first to assess in-the-moment predictors of risky drinking in the postpartum period and will thus fill critical gaps in existing research. New mothers who engage in risky drinking and other substance use are understudied and underserved as much of the intervention research on perinatal substance use is focused on pregnancy despite high risks of increasing substance use in the early postpartum weeks [5].

The study findings will enhance our understanding of daily and momentary fluctuations in risk and protective factors for risky drinking during the early postpartum period, a time when risk for alcohol use is high and access to treatment is often low [5]. Although there is substantial theoretical and empirical literature on the risk and protective factors for risky drinking in the general adult population [53,151,152], this study will be the first to examine whether established models apply to the unique population of new mothers. In addition, the study findings will elucidate the role of maternal-specific factors such as baby fussiness and maternal self-efficacy in postpartum risky drinking. Very little is currently known about alcohol use risk during the early postpartum weeks, and data gleaned from this study will provide the information needed to develop tailored interventions for this underserved and high-risk population.

### Strengths and Limitations

A primary strength of this study is the reliance on theory to guide EMA data collection and JITAI development. A recent systematic review of JITAIs for substance use found that most existing studies did not apply state-of-the-art methods such as the MOST framework and did not sufficiently incorporate theory into intervention development [153]. The base of empirical studies on brief interventions for postpartum risky drinking is extremely small, and those studies that do exist have not adequately incorporated theoretically driven behavior change techniques [154]. Thus, the JITAI to ultimately be developed in this study stands to significantly improve upon existing interventions by including specific behavior change techniques that are clearly mapped onto theory. Studies show that TMIs that integrate behavior change principles and are adaptively tailored generate larger effects on clinical outcomes [20,33].

The inclusion of variables that are especially salient in the postpartum period, such as maternal self-efficacy, baby irritability, and sleep, is an additional strength of this study. Studies of mothers in substance use treatment demonstrate a complex relationship between motherhood and substance use treatment and recovery. Although motherhood and caring for children are often described as a critical motivating factor for seeking treatment and reducing substance use [42,155], mothers are also more likely to conceal their substance use and avoid seeking help because of fears of losing their children [156,157]. Loss of child custody may also lead to relapse in mothers because of the stress and trauma of child removal [158,159]. Given these complex relationships, assessing variables related to the mother’s role, such as maternal self-efficacy, is critical for the appropriate tailoring of interventions.

Study limitations include the requirement to speak and read English and own a smartphone with internet access, limiting generalizability. In addition, the study is focused on alcohol use only, which may leave needs related to the use of other substances unaddressed. Many pregnant and postpartum people who engage in risky drinking also use other substances [160,161]. Finally, participant noncompliance and attrition is often a limitation of EMA studies [57]. Although the few previous EMA studies that have been conducted with new mothers report compliance rates of 75%-80% [106,131], one of the goals of this study is to assess the feasibility of the EMA protocol in this understudied population to inform future research.

### Conclusions and Future Directions

The need for tailored digital supportive interventions for the postpartum period is greater than ever given the increasing rates of perinatal stress, depression, and substance use during the COVID-19 pandemic [162]. A growing number of studies have shown increases in postpartum anxiety and depression, perinatal stress, and difficulties with bonding and breastfeeding since the beginning of the pandemic [163,164], all of which significantly increase the risk for risky drinking. This increased risk is combined with the fact that women who use substances are less likely to receive postpartum care [165] and are more likely to report poor relationships with their health care providers and negative experiences seeking care [166]. Digital interventions are generally acceptable to new mothers as a way of receiving support for risky drinking and other behavioral health concerns [25,167] and have the potential to fill a significant gap in services that is currently being exacerbated by the pandemic.

Despite the limitations, this study has the potential to significantly contribute to the existing literature by improving our understanding of the antecedents of postpartum risky drinking and informing the development of a tailored JITAI to address it. If feasibility is supported, the EMA protocol can also serve as a model for future studies that aim to collect real-time data from new mothers. This study represents a first step in a larger program of research aimed at using technology to reach underserved new mothers with interventions for perinatal substance use that are evidence-based and tailored to their identities as mothers and aim to empower mothers to seek help while reducing stigma and fear. The methods and findings of this study will be applied to future efforts to ultimately expand the JITAI to include other substances beyond alcohol as well as to create culturally tailored versions. Significant racial and ethnic disparities exist in access to support and treatment for substance use, with Black and Latinx mothers experiencing higher levels of stigma and greater access barriers to obtaining needed support [168,169]. An important future direction for our research program is to partner with Black and Latinx communities to develop theory-driven, tailored, and technology-based interventions to better meet the needs of Black and Latinx new mothers.

## Appendix

Multimedia Appendix 1 National Institute on Alcohol Abuse and Alcoholism grant reviews.

## Abbreviations

CI: Central Intake
EMA: ecological momentary assessment
JITAI: just-in-time adaptive intervention
MOST: multiphase optimization strategy
TMI: text messaging intervention
