# Supplementary material for: Adaptive Text Messaging for Postpartum Risky Drinking: Conceptual Model and Protocol for an Ecological Momentary Assessment Study
Source: JMIR Res Protoc. 2022 Apr 4;11(4):e36849. doi: 10.2196/36849 (PMC9016512; doi:10.2196/36849)
Supplement: Multimedia Appendix 1 [file resprot_v11i4e36849_app1.pdf]

SUMMARY STATEMENT
(Privileged Communication)

PROGRAM CONTACT:
BRETT HAGMAN
301-443-0638
brett.hagman@nih.gov

Release Date: **06/22/2020**
Revised Date:

Application Number: **1 R34 AA028407-01A1**
Formerly: **1R34AA028407-01**

Principal Investigator

DAUBER, SARAH E.

Applicant Organization: NATIONAL CENTER ON ADDICTION/SUB ABUSE

Review Group: **IPTA**
Interventions to Prevent and Treat Addictions Study Section

Meeting Date: **06/04/2020**
Council: **OCT 2020**
Requested Start: **12/01/2020**

RFA/PA: **PAR19-213**
PCC: **AC H**
Dual PCC: **CC/KLF**
Dual IC(s): **DA**

Project Title: **Adaptive Text Messaging Intervention for Risky Drinking in Postpartum Women**

SRG Action: **Impact Score:25**
Next Steps: **Visit https://grants.nih.gov/grants/next_steps.htm**
Human Subjects: **30-Human subjects involved - Certified, no SRG concerns**
Animal Subjects: **10-No live vertebrate animals involved for competing appl.**
Gender: **2A-Only women, scientifically acceptable**
Minority: **1A-Minorities and non-minorities, scientifically acceptable**
Age: **7A-Only Adults, scientifically acceptable**

Project Year	Direct Costs Requested	Estimated Total Cost
1	150,000	251,012
2	150,000	251,012
3	150,000	251,012
TOTAL	450,000	753,037

ADMINISTRATIVE BUDGET NOTE: The budget shown is the requested budget and has not been adjusted to reflect any recommendations made by reviewers. If an award is planned, the costs will be calculated by Institute grants management staff based on the recommendations outlined below in the COMMITTEE BUDGET RECOMMENDATIONS section.

1R34AA028407-01A1 DAUBER, SARAH

RESUME AND SUMMARY OF DISCUSSION: This application proposes to use the Multiphase Optimization Strategy (MOST) to develop and test a text messaging intervention for risky drinking in post-partum women. Reviewers agreed about the potential significance of developing a disseminable intervention to address the high rates of risky drinking and associated problems in post-partum women. The resubmission retains the original strengths including the innovative use of MOST to tailor intervention components to the unique concerns of post-partum women, the well-qualified investigators, and the rigorous ecological momentary assessment (EMA) measures and analyses. The investigators were highly responsive to the prior review, including collecting data to support feasibility and adjusting the aims to focus more on feasibility than efficacy. While the conceptual framework was strengthened, there was a lingering concern about the application of the relapse prevention model. Reviewers also noted concerns with the approach, including the limited selection of relapse triggers, insufficient justification for the 6-week post-partum timing, and lack of consideration of the context surrounding the woman (e.g. social support). Following the discussion, however, the panel concluded that the application's strengths outweigh its weaknesses and the project's potential impact is high.

DESCRIPTION (provided by applicant): The proposed R34 will develop a theory-driven, adaptive text messaging intervention (TMI) for risky drinking in postpartum women. Risky drinking is prevalent among women of reproductive age, and more than half of women who reduce their alcohol use during pregnancy return to pre-pregnancy levels by three months postpartum. Postpartum risky drinking can impair a mother's ability to care for her baby and is associated with increased risk for child abuse and neglect and involvement with the child welfare system, as well as poor long- term child outcomes. Most adults who engage in risky drinking do not seek treatment, and postpartum women may be especially unlikely to seek formal treatment due to stigma and fears of child protective services involvement. Text messaging is a promising strategy for reaching non-treatment-seeking risky drinkers that may be particularly suitable for postpartum women, as it is convenient and anonymous. This approach also has wide scalability potential, as it is capable of reaching large numbers of people at relatively low cost. The proposed study will apply the Multiphase Optimization Strategy (MOST) framework, an engineering-inspired approach for developing efficient and scalable mobile health interventions, to develop the first adaptive TMI specifically designed for risky drinking in postpartum women. The proposed study will refine a theoretical model to inform the TMI that is based on the Relapse Prevention model and specifies the temporal relations of stress, negative mood, and maternal self-efficacy with intention to drink and daily drinking behavior in postpartum women, and their daily fluctuations in response to triggers that are unique to the postpartum period (sleep quality and baby irritability). The proposed TMI will include behavior change interventions based on motivational interviewing and cognitive behavioral therapy that target coping skills and motivation to avoid drinking, and that are adaptively tailored to fluctuations in stress, negative mood, and self-efficacy. Specific aims are to (1) test and refine the proposed theoretical model via Ecological Momentary Assessment with 30 women for 14 days to determine daily fluctuations in stress, negative mood, and self-efficacy in response to triggers and their impact on drinking intentions; (2) develop the content and decision rules for the TMI via iterative cycles of user feedback collected via focus groups with 30 postpartum women; and (3) conduct a pilot micro-randomized trial (MRT) with 50 women to assess feasibility of design and methods and obtain preliminary proof-of-concept data to prepare for a future fully-powered optimization trial. Study products will be a set of preliminary TMI components for postpartum risky drinking, ready for optimization and evaluation in a future larger scale R01, and a set of piloted EMA and MRT methods for the population of postpartum risky drinkers.

PUBLIC HEALTH RELEVANCE: Risky drinking in postpartum women is a critical public health problem that can interfere with a mother's ability to care for her baby, and is associated with increased risk for child abuse and neglect and poor long-term child health and developmental outcomes. Postpartum women are often not connected to formal service systems, and often do not receive needed help for risky drinking due to the stigma associated with substance use in mothers and fears of child protective services involvement if they disclose alcohol use to a professional. The proposed R34 will develop and test an innovative text messaging intervention to address risky drinking in postpartum women.

CRITIQUE 1

Significance: 2
Investigator(s): 2
Innovation: 2
Approach: 2
Environment: 3

Overall Impact: Over a quarter of postpartum women report risky drinking. This R34 project will develop a self-efficacy enhancing and Relapse Prevention model-based Text Messaging Intervention tailored for postpartum women with risky drinking. Investigators are strong, with complementary expertise, and longstanding partnerships with a New Jersey child abuse prevention network providing care for a large number of postpartum women. Factors in the approach that improve the overall score include 1) good access to the target population that will facilitate accruing the sample needed for formative and pilot trial work needed to complete this 3 year project, 2) a logical series of formative work with 30 women from the target population including EMA testing, focus groups, interviews, and user centered design work to develop the text messaging intervention, 3) the intervention will be built on an existing framework already developed by co-I Muensch, and 4) feasibility, acceptability, and effect sizes of risky drinking reductions will be determined in a 6 week micro-randomized trial design with 50 postpartum women with risky drinking. A weakness in the approach is inadequate detail about pre-recruitment of women during the last trimester of pregnancy (given that they may not be reporting risky drinking at that time). The primary innovation is that this would be the first text messaging intervention targeting risky drinking among postpartum women, an underserved group whose drinking could cause harm to self and/or baby. A weakness in innovation is that text messaging is certainly not novel. Timeline is reasonable and proposed project benchmarks seem achievable. Overall, this is a strong proposal with several minor weaknesses.

1. Significance:

Strengths

- This resubmitted proposal will develop, and feasibility test a new theory-based text messaging intervention to reduce problem drinking among mothers in the postpartum period in the age range of 18-45
- The proposal appears to be highly responsive to prior critique, with many improvements to the theoretical model, approach, investigator time, and feasibility measurements
- Proposal shows evidence that postpartum drinking among mothers is common (26% were risky drinkers)
- This project could have a significant impact, and the proposal shows evidence that text messaging intervention is highly scalable, feasible and acceptable among risky drinkers, and

show evidence of impact on related behaviors in prior studies with postpartum women (smoking, cannabis use, depression)

Weaknesses

- (Minor) No previous evidence that postpartum women have responded to text messaging interventions with actual drinking reductions
- Proposal cites limited evidence that postpartum drinking relates to specific harms

2. Investigator(s):

Strengths

- PI Dauber is well prepared to oversee the study
- Very strong team of experts with different roles articulated in budget justification
- PI and quantitative data analyst effort acceptable
- Separate qualitative analyst West will guide interpretation of formative qualitative data
- Consultant time reasonable, increased from prior proposal

Weaknesses

- None noted

3. Innovation:

Strengths

- This would be the first Text Messaging intervention to address postpartum risky drinking
- Designs such as MOST and micro-randomized trials are underutilized at present and increase the innovation of the project

Weaknesses

- Text messaging itself is not novel.

4. Approach:

Strengths

- Proposal will build on Co-I Muench's Text Messaging intervention (TMI) instead of CBPR or Text4Baby. That intervention for adult drinking provides the proposed study with EMAs that predicted real time drinking and an existing message bank about drinking.
- The intervention rationale is that triggers unique to the postpartum period such as sleep deprivation and baby irritability can lead to increased stress and negative mood, along with decreased self-efficacy. Theoretical basis of intervention to be developed is the drinking Relapse Prevention Model. The investigators include a proposed causal chain from stressors and coping mechanisms to drinking behavior. Relatedly, motivation and coping are now targets of the TMI. The rigor of previous work is mixed, with ½ of tested TMIs reducing alcohol use or increasing readiness to change, but studies were of low to moderate quality.
- Several important enhancements since prior proposal include:

- 1). New feasibility data gathered since last application from a survey of postpartum women showed an adequate sample of risky drinkers willing to consider participation, and an adequate number of texters.
- 2). Feasibility and acceptability metrics will include: time to recruit participants, demographics and clinical characteristics of enrollees vs. decliners, and engagement metrics such as the number of EMA responses, messages viewed, and days engaged with the TMI, with retention measured with multilevel mixed effects models
- 3). Qualitative analysis processes and outcomes are adequately described
- 4) Standard measures of addiction severity, mental disorders, parenting stress, maternal self-efficacy, and postnatal depression will be used
- Aim 1 will focus on testing EMA for messaging triggers and reports of risk factors, to determine optimal tailoring messages and decision rules. EMA during this phase will determine if it is feasible and acceptable, and data generated will show dynamic relationships between risks and behaviors to guide optimization of text message volume and timing. Data analysis plan for this aim is fully described.
- Aim 2 will focus on applying principles of user centered design and testing drafts of message content, language, tone, and decision rules with 30 women in several focus groups. Additionally, they will pilot the draft TMI for 5 days to give final feedback. These data will be analyzed for core themes.
- Strong existing relationships with recruitment sites within the Prevent Child Abuse New Jersey network lend confidence that recruitment of adequate participants will be successful; plans are in place to reach beyond Essex county if needed.
- Using the multiphase optimization process and micro-randomized trials increase power to determine intervention components' impact in an efficient design
- Aim 3 is a micro randomized trial in which randomization will occur at each decision point for each of the 50 participants. Practical matters are well specified and carefully thought out. Strengths include conducting baselines in person or on phone, completing a trial of 2 text messages to consider it a completed enrollment, and planning a 6 week intervention period with text messaged assessments culminating in 126 randomizations per participant. The impact of the MRT phase of the project on actual drinking will be evaluated using effect sizes, appropriate for a preliminary study.
- Overall, the MRT will provide data on intervention feasibility and acceptability, with preliminary evaluation of potential main effects on drinking

Weaknesses

- Recruitment of women during pregnancy may result in over-recruitment of lower risk drinking women. Women will be recruited during the last trimester of pregnancy, when they are less likely to be drinking. Unclear how many of these will resume risky drinking and how eligibility will be confirmed upon enrollment which is planned for a time very close to the birth. How many women will immediately resume risky drinking? Data provided do not really address this timing issue. Recruitment flow and tracking of women during the last trimester generally could be more thoroughly described.

5. Environment:

Strengths

- The Center on Addiction has a strong track record of sponsored research, and has developed collaborative relationships with nearby universities and services providers. It has library and technology resources. Its strengths, including the existing cloud-based text messaging platform, seem similar to those at some universities.

Weaknesses

- The Center lacks the full array of services hosted by most universities.

Study Timeline:

Strengths

- There is a detailed timeline provided by month and milestone.
- Investigators have added more time to complete Aims 1 and 2. Each phase will be completed in approximately 10-11 months.

Weaknesses

- None noted

Protections for Human Subjects:

Acceptable Risks and/or Adequate Protections

- Investigators have included plans to coach participants on securing their phones, deleting text messages, and will include a COC

Data and Safety Monitoring Plan (Applicable for Clinical Trials Only):

Acceptable

- Inclusion of COC strengthens protections

Inclusion Plans:

- Sex/Gender: Distribution justified scientifically
- Race/Ethnicity: Distribution justified scientifically
- For NIH-Defined Phase III trials, Plans for valid design and analysis: Not applicable
- Inclusion/Exclusion Based on Age: Distribution justified scientifically

Vertebrate Animals:

Not Applicable (No Vertebrate Animals)

Biohazards:

Not Applicable (No Biohazards)

Resubmission:

- Proposal seems highly responsive to prior critiques. Most prior concerns have been addressed thoughtfully.

Resource Sharing Plans:

Acceptable

Budget and Period of Support:
Recommend as Requested

CRITIQUE 2

Significance: 3
Investigator(s): 1
Innovation: 1
Approach: 6
Environment: 1

Overall Impact: This is an R34 resubmission in response to PAR19-213. The application proposes to develop a theory-driven, adaptive text messaging intervention (TMI) for risky drinking in postpartum women. The study will apply the Preparation Phase of the Multiphase Optimization Strategy (MOST) framework to develop and refine a TMI that is based the Relapse Prevention model. Based on preliminary research, the triggers of prior night sleep and baby irritability are thought to increase stress, negative mood, and self-efficacy, which, in turn, impact drinking behavior. TMI intervention content based on motivational interviewing and cognitive behavioral therapy might mitigate this causal chain. Specific aims include (1) using Ecological Momentary Assessment to test and refine the proposed theoretical model; (2) developing content and decision rules for the TMI through focus group methodology; and (3) conducting a pilot micro-randomized trial (MRT) to assess feasibility and preliminary data to power a larger trial. In MRT, participants are re-randomized throughout the trial, allowing for the examination of causal time-varying effects of intervention components on proximal outcomes with a smaller number of participants. The application creates strong scientific premise for developing TMI interventions with postpartum women and includes data on the feasibility of the study methods. The investigative team is strong with supportive environments. The methods are innovative. The approach is generally rigorous and is organized by a strong theoretical model, however, there are some concerns about the application of Relapse Prevention as a theoretical model, the inclusion of only a limited set of triggers (identified via correlational research) and intervention components, a focus on abstinence (vs. harm reduction) that seems incongruent with a risky drinking intervention, and concerns about the possible heterogeneity of the sample in terms of drinking severity.

1. Significance:

Strengths

- The application synthesizes research demonstrating that risky drinking is prevalent among post-partum women and that risky drinking can negatively impact child outcomes, creating a rationale for the development of effective interventions with this population.
- The application also demonstrates that individuals who engage in risky drinking as well as postpartum women are unlikely to seek traditional treatment creating a rationale for the promise of text messaging interventions.
- TMIs for other perinatal health behaviors, including some by members of the investigative team, have shown promise.
- The application highlights research suggesting the TMIs should be theory-driven, incorporate evidence-based interventions, and adapt to in-the-moment fluctuations in risk factors.

- The team collected feasibility data demonstrating that the population of interest can receive text messages and a large proportion would be willing to participate and receive messages.

Weaknesses

- In some cases, the studies that are cited to create rationale for the current project, are provided in insufficient detail to evaluate their rigor.
- There are some concerns about the selection of relapse prevention as the guiding theoretical model. RP is a type of CBT and has some misalignment to risky drinking, for which interventions are focused more on reducing risk than abstinence. RP is used as the guiding theoretical model, while CBT and MI are framed as intervention approaches, but all three are models of behavior change with corresponding intervention approaches.

2. Investigator(s):

Strengths

- The PI has a track record of research focused on improving access to substance abuse and mental health treatment for pregnant and parenting women, with a body of relevant publications and several NIH grant leadership experiences.
- The relevant expertise and roles and responsibilities of Co-Is and consultants are clearly defined and include a focus on qualitative research (West), EMA (Thurl), digital adaptive interventions (Muench), motivational interviewing (Ondersma), MOST Framework (Guastaferro), and MRTs (Murphy).

Weaknesses

- None noted.

3. Innovation:

Strengths

- There is no existing TMI for risky drinking that is specifically designed for postpartum women.
- EMA will provide event level data for measured factors that is not currently understood for postpartum women.
- The use of MRTs is innovative.

Weaknesses

- Note noted.

4. Approach:

Strengths

- The approach to collect data appears feasible and rigorous.
- The Multiphase Optimization Strategy (MOST) framework provides clear and organized guidance on developing TMIs.
- Eligibility criteria and recruitment procedures are clearly described.
- Instrumentation to measure triggers, proximal mediators, and proximal outcomes is clearly described

- Feasibility measures are clearly described.
- Analytic strategies are clearly described and appear appropriate to meet the aims.

Weaknesses

- While there is some rationale provided to demonstrate the likely impact of the selected triggers, it is not clear why triggers necessarily need to be specific to the postpartum period as other factors that affect the larger population of risky drinkers (e.g. social triggers) may have a larger impact.
- It is not clear how the study will proceed if Aim 1 does not reveal a relationship between the proposed stressors and drinking. It appears Aim 1 assumes that these triggers will be an important part of the model and this may not be the case. A more conservative approach might test a larger sample of triggers in Aim 1 and select those that are most predictive.
- The discussion of specific intervention components to be explored is a little underdeveloped, particularly for MI. None of the intervention components focus on the relational aspect of MI or strategically evoking motivation. The 3 suggested components could fit in an MI model, but could equally fit in a CBT model. Like above, it might make more sense to provide participants with a larger pool of intervention components to explore.
- Women who drink regularly during pregnancy are likely more severe than women who exceed recommended drinking limits pre-pregnancy. The inclusion criteria allows for both groups who may respond differently to the intervention. It may be appropriate to have a severity ceiling given that the intervention is focused on risky drinking (vs. alcohol use disorder).
- Given that the intervention is targeting risky drinking, it seems appropriate that the target of the intervention is reduced drinking or harm reduction (versus abstinence). It is not clear whether the intervention content will focus on decreased drinking or abstinence. The RP model is definitely more appropriate for abstinence. Outcome variables, including motivation to abstain and 7-day point prevalence abstinence also seem like a mismatch, given the study population.
- If intervention content is adaptively tailored to fluctuations in stress, negative mood, and self-efficacy it is not clear what type of intervention content will be sent if stressors and proximal outcomes are all low.
- There are concerns about assessment reactivity given the extensive baseline assessment for a relatively minimal intervention.

5. Environment:

Strengths

- The National Center on Addiction and Substance Abuse and Johns Hopkins University have the resources necessary to support the aims of the grant.
- The PI's partnership with Prevent Child Abuse New Jersey will facilitate recruitment of the sample.

Weaknesses

- None noted.

Study Timeline:

Strengths

- The timeline is very ambitious but appears feasible and appropriate to meet the aims of the grant.

Weaknesses

- None noted by reviewer.

Protections for Human Subjects:

Acceptable Risks and/or Adequate Protections
Data and Safety Monitoring Plan (Applicable for Clinical Trials Only):
Acceptable

Inclusion Plans:

- Sex/Gender: Distribution justified scientifically
- Race/Ethnicity:
- For NIH-Defined Phase III trials, Plans for valid design and analysis:
- Inclusion/Exclusion Based on Age:
- The sample will include female adults that are representative of the ethnic/racial makeup of the community.

Vertebrate Animals:

Not Applicable (No Vertebrate Animals)

Biohazards:

Not Applicable (No Biohazards)

Resubmission:

- The resubmission was highly responsive to previous reviewers' concerns. The application provided additional research to provide scientific premise for the current study and adopted a new conceptual model to guide the TMI. The team also collected data to support feasibility. In addition, the aims have been adjusted to focus more on feasibility than efficacy.

Resource Sharing Plans:

Not Applicable (No Relevant Resources)

Budget and Period of Support:

Recommend as Requested

CRITIQUE 3

Significance: 2
Investigator(s): 2
Innovation: 2

Approach: 2
Environment: 1

Overall Impact: This proposal addresses a very significant issue in the field of postpartum risky drinking and overall impact is potentially high because the research is likely to provide insight into proximal predictors of postpartum drinking along with the development of a TMI that may reduce risky postpartum drinking. The project is innovative, and the approach has some very strong aspects such as the EMA to test the theory driving the TMI development and the focus groups to inform TMI development. Most of the weaknesses were minor. The investigative team seems appropriate to conduct the proposed work.

1. Significance:

Strengths

- In other populations (college students), daily variability in stress and negative affect, as opposed to average levels, were significantly related to alcohol consumption, and alcohol-related problems, that may vary throughout the day. Associations between daily fluctuations in negative affect and alcohol craving and quantity have also been found. When considered in light of data indicating a relation between depression and drinking among post-partum women, investigators provide solid rationale for an intervention to help post-partum women cope with such fluctuations in mood associated with the post-partum period
- Theoretical model that informs the intervention appears based on extant research that supports each pathway
- Given that postpartum women may be reluctant to seek treatment for risky drinking (due to fear of implications for baby and/or limited ability to seek treatment while responsible for child care), efforts to improve interventions that can be completed at home at the patient's convenience are especially important, with high potential for impact.
- Just in time tailoring may improve impact

Weaknesses

- Authors report that prior studies of TMI demonstrate that some of them can reduce drinking yet the magnitude of these decreases is not reported, limiting ability to predict the impact of the proposed study

2. Investigator(s):

Strengths

- The PI has several years' experience on addictions related research and has been PI on 3 NIH funded projects (with several others as Co-I etc.). She has experience conducting substance-related and mood-related research with post-partum women.
- The investigative team brings complementary expertise necessary for the successful completion of the proposed project.

Weaknesses

- The PI has relatively few first authored manuscripts (8) given that she graduated with her PhD in 2004

3. Innovation:

Strengths

- Investigators state that TMIs have demonstrated empirical support for reducing risky drinking in nontreatment-seeking adults, yet there are no studies of TMIs for alcohol use in postpartum women; thus, the proposed study will be the first TMI for postpartum risky drinking.
- Theory driven TMI to inform MET-CBT to be used is innovative
- The use of a just-in-time adaptive TMI for postpartum risky drinking is innovative
- EMA data will provide information on the dynamic relations among stress, negative mood, maternal self-efficacy and drinking and daily fluctuations in these relations in postpartum women, which has thus far been understudied.

Weaknesses

- None noted

4. Approach:

Strengths

- Pilot data to test theoretical model's utility in predicting risky drinking among postpartum women an important step that will inform the intervention's development and feasibility of EMA with this population
- Several strategies will be used to improve compliance with EMA protocol
- Aims for focus groups seem appropriate to inform TMI development

Weaknesses

- Investigators state that the intervention will run until 6 weeks postpartum given that 6 weeks also coincides with the time of highest risk during the postpartum period, as maternal postpartum distress peaks around 6 weeks after delivery; yet no data are provided to support that weeks 1-6 are associated with risky drinking among postpartum women.

5. Environment:

Strengths

- The Center on Addiction has the resources necessary to conduct the proposed project.

Weaknesses

- None

Study Timeline:

Strengths

- Seems appropriate

Weaknesses

- None

Protections for Human Subjects:

Acceptable Risks and/or Adequate Protections

- Appropriate plans in place to protect participant and infant safety

Data and Safety Monitoring Plan (Applicable for Clinical Trials Only):
Unacceptable

- No information regarding the DSMB

Inclusion Plans:

- Sex/Gender: Distribution justified scientifically
- Race/Ethnicity: Distribution justified scientifically
- For NIH-Defined Phase III trials, Plans for valid design and analysis:
- Inclusion/Exclusion Based on Age: Distribution justified scientifically
- Participants will be women aged 18-45. Racial/ethnic composition to reflect community of study.

Vertebrate Animals:
Not Applicable (No Vertebrate Animals)

Biohazards:
Not Applicable (No Biohazards)

Resubmission:

- Investigators appear responsive to original reviews.

Resource Sharing Plans:
Acceptable

- Plans to share results and data.

Budget and Period of Support:
Recommend as Requested

THE FOLLOWING SECTIONS WERE PREPARED BY THE SCIENTIFIC REVIEW OFFICER TO SUMMARIZE THE OUTCOME OF DISCUSSIONS OF THE REVIEW COMMITTEE, OR REVIEWERS' WRITTEN CRITIQUES, ON THE FOLLOWING ISSUES:

PROTECTION OF HUMAN SUBJECTS: ACCEPTABLE

INCLUSION OF WOMEN PLAN: ACCEPTABLE

INCLUSION OF MINORITIES PLAN: ACCEPTABLE

INCLUSION ACROSS THE LIFESPAN: ACCEPTABLE

COMMITTEE BUDGET RECOMMENDATIONS: The budget was recommended as requested.

Footnotes for 1 R34 AA028407-01A1; PI Name: DAUBER, SARAH E.

NIH has modified its policy regarding the receipt of resubmissions (amended applications).See Guide Notice NOT-OD-18-197 at https://grants.nih.gov/grants/guide/notice-files/NOT-OD-18-197.html. The impact/priority score is calculated after discussion of an application by averaging the overall scores (1-9) given by all voting reviewers on the committee and multiplying by 10. The criterion scores are submitted prior to the meeting by the individual reviewers assigned to an application, and are not discussed specifically at the review meeting or calculated into the overall impact score. Some applications also receive a percentile ranking. For details on the review process, see http://grants.nih.gov/grants/peer_review_process.htm#scoring.

MEETING ROSTER

Interventions to Prevent and Treat Addictions Study Section
Risk, Prevention and Health Behavior Integrated Review Group
CENTER FOR SCIENTIFIC REVIEW
IPTA
06/04/2020 - 06/05/2020

Notice of NIH Policy to All Applicants: Meeting rosters are provided for information purposes only. Applicant investigators and institutional officials must not communicate directly with study section members about an application before or after the review. Failure to observe this policy will create a serious breach of integrity in the peer review process, and may lead to actions outlined in NOT-OD-14-073 at https://grants.nih.gov/grants/guide/notice-files/NOT-OD-14-073.html and NOT-OD-15-106 at https://grants.nih.gov/grants/guide/notice-files/NOT-OD-15-106.html, including removal of the application from immediate review.

CHAIRPERSON(S)

WALTON, MAUREEN A, PHD, MPH
PROFESSOR
DEPARTMENT OF PSYCHIATRY
UNIVERSITY OF MICHIGAN
ANN ARBOR, MI 48109

MEMBERS

ALESSI, SHEILA MARIE, PHD *
ASSOCIATE PROFESSOR
DEPARTMENT OF PSYCHIATRY
CALHOUN CARDIOLOGY CENTER
UNIVERSITY OF CONNECTICUT HEALTH CENTER
FARMINGTON 06030

BERKEL, CADY, PHD *
ASSOCIATE RESEARCH PROFESSOR
REACH INSTITUTE
SOUTHWEST INTERDISCIPLINARY RESEARCH CENTER
ARIZONA STATE UNIVERSITY
TEMPE, AZ 85284

BERMAN, MITCHELL E, PHD *
PROFESSOR AND DEPARTMENT HEAD
DEPARTMENT OF PSYCHOLOGY
MISSISSIPPI STATE UNIVERSITY
MISSISSIPPI STATE, MS 39762

BERNSTEIN, STEVEN L, MD
PROFESSOR AND VICE CHAIR
DEPARTMENT OF EMERGENCY MEDICINE
YALE UNIVERSITY SCHOOL OF MEDICINE
NEW HAVEN, CT 06519

BRADIZZA, CLARA M, PHD
PROFESSOR
SCHOOL OF SOCIAL WORK
UNIVERSITY AT BUFFALO
STATE UNIVERSITY OF NEW YORK
BUFFALO, NY 14203

BUCKNER, JULIA D, PHD *
PROFESSOR AND DIRECTOR OF CLINICAL TRAINING
DEPARTMENT OF PSYCHOLOGY
LOUISIANA STATE UNIVERSITY
BATON ROUGE, LA 70803

COLEMAN-COWGER, VICTORIA HOPE, PHD
CLINICAL RESEARCH DIRECTOR
THE EMMES CORPORATION
ROCKVILLE, MD 20850

CROPSEY, KAREN L, PSYD
PROFESSOR
DEPARTMENT OF PSYCHIATRY AND BEHAVIORAL NEUROLOGY
UNIVERSITY OF ALABAMA AT BIRMINGHAM
BIRMINGHAM, AL 35294

DUNN, KELLY E., PHD *
ASSOCIATE PROFESSOR
DEPARTMENT OF PSYCHIATRY AND BEHAVIORAL SCIENCES
SCHOOL OF MEDICINE
JOHNS HOPKINS UNIVERSITY
BALTIMORE, MD 21224-6823

FRIEDMANN, PETER D, MD, MPH
PROFESSOR
DEPARTMENT OF MEDICINE
UNIVERSITY OF MASSACHUSETTS
MEDICAL SCHOOL - BAYSTATE
SPRINGFIELD, MA 01107

GRANT, SEAN, PHD *
ASSISTANT PROFESSOR
RICHARD M. FAIRBANKS SCHOOL OF PUBLIC HEALTH
INDIANA UNIV-PURDUE UNIV AT INDIANAPOLIS
INDIANAPOLIS, IN 46202

GRYCZYNSKI, JAN, PHD
SENIOR RESEARCH SCIENTIST
FRIENDS RESEARCH INSTITUTE
BALTIMORE, MD 21201

HETTEMA, JENNIFER ELIN, PHD *
ASSOCIATE PROFESSOR
DEPARTMENT OF FAMILY
AND COMMUNITY MEDICINE
UNIVERSITY OF NEW MEXICO SCHOOL OF MEDICINE
ALBUQUERQUE 87131

HILL, KEVIN P., MD *
ASSOCIATE PROFESSOR OF PSYCHIATRY
HARVARD MEDICAL SCHOOL
BOSTON, MA 02215

HITSMAN, BRIAN L, PHD
ASSOCIATE PROFESSOR
DEPARTMENT OF PREVENTIVE MEDICINE
FEINBERG SCHOOL OF MEDICINE
NORTHWESTERN UNIVERSITY
CHICAGO, IL 60611

INGERSOLL, KAREN S, PHD *
PROFESSOR
DEPARTMENT OF PSYCHIATRY
AND NEUROBEHAVIORAL SCIENCES
UNIVERSITY OF VIRGINIA
CHARLOTTESVILLE, VA 22911

KAHLER, CHRISTOPHER W., PHD *
PROFESSOR AND CHAIR
DEPARTMENT OF BEHAVIORAL
AND SOCIAL SCIENCES
CENTER FOR ALCOHOL AND ADDICTION STUDIES
BROWN UNIVERSITY SCHOOL OF PUBLIC HEALTH
PROVIDENCE, RI 02912

KIM-MOZELESKI, JIN E., PHD *
ASSISTANT PROFESSOR
DEPARTMENT OF POPULATION AND
QUANTITATIVE HEALTH SCIENCES
CASE WESTERN RESERVE UNIVERSITY
CLEVELAND, OH 44106

LARSON, MARY JO, PHD
SENIOR SCIENTIST
INSTITUTE FOR BEHAVIORAL HEALTH
HELLER SCHOOL FOR SOCIAL POLICY AND MANAGEMENT
BRANDEIS UNIVERSITY
WALTHAM, MA 02454

LITT, DANA M, PHD *
ASSOCIATE PROFESSOR
DEPARTMENT OF HEALTH BEHAVIOR AND HEALTH
SYSTEMS
SCHOOL OF PUBLIC HEALTH SYSTEMS
UNIVERSITY OF NORTH TEXAS
FORT WORTH 76107

LOVEJOY, TRAVIS IAN, MPH, PHD *
ASSOCIATE PROFESSOR
DEPARTMENT OF PSYCHIATRY
SCHOOL OF MEDICINE
OREGON HEALTH AND SCIENCE UNIVERSITY
PORTLAND, OR 97239

MAHABEE-GITTENS, E. MELINDA, MD
PROFESSOR
DIVISION OF EMERGENCY MEDICINE
CINCINNATI CHILDREN'S HOSPITAL MEDICAL CENTER
CINCINNATI, OH 45229

MAISTO, STEPHEN A, PHD *
PROFESSOR
DEPARTMENT OF PSYCHOLOGY
SYRACUSE UNIVERSITY
SYRACUSE, NY 13244

MCGOVERN, MARK P, PHD
PROFESSOR
DEPARTMENT OF PSYCHIATRY
AND BEHAVIORAL SCIENCES
STANFORD UNIVERSITY
PALO ALTO, CA 93404

MENDELSON, TAMAR, PHD
PROFESSOR
DEPARTMENT OF MENTAL HEALTH
JOHNS HOPKINS BLOOMBERG SCHOOL OF PUBLIC HEALTH
BALTIMORE, MD 21205

MILLER, MARY ELIZABETH, PHD *
ASSISTANT PROFESSOR
DEPARTMENT OF PSYCHIATRY
UNIVERSITY OF MISSOURI
COLUMBIA, MO 65211

MONTGOMERY, LATRICE, PHD *
ASSISTANT PROFESSOR
ADDICTION SCIENCES DIVISION
DEPARTMENT OF PSYCHIATRY
AND BEHAVIORAL NEUROSCIENCE
UNIVERSITY OF CINCINNATI COLLEGE OF MEDICINE
CINCINNATI, OH 45229

NOONAN, DEVON, PHD *
ASSOCIATE PROFESSOR
SCHOOL OF NURSING
DUKE UNIVERSITY
DURHAM, NC 27710

OLIVETO, ALISON, PHD
PROFESSOR AND VICE CHAIR FOR RESEARCH
DEPARTMENT OF PSYCHIATRY
UNIVERSITY OF ARKANSAS FOR MEDICAL SCIENCES
LITTLE ROCK, AR 72205

OSILLA, KAREN C, PHD
SENIOR BEHAVIORAL SCIENTIST
RAND CORPORATION
SANTA MONICA, CA 90404

PARROTT, DOMINIC, PHD *
PROFESSOR
DEPARTMENT OF PSYCHOLOGY
GEORGIA STATE UNIVERSITY
ATLANTA, GA 30302

PIPER, MEGAN E, PHD
ASSOCIATE PROFESSOR
DEPARTMENT OF MEDICINE
UNIVERSITY OF WISCONSIN-MADISON
MADISON, WI 53711

SCHEUERMANN, TANEISHA SHANI, PHD *
ASSISTANT PROFESSOR
DEPARTMENT OF PREVENTIVE MEDICINE AND
PUBLIC HEALTH
UNIVERSITY OF KANSAS MEDICAL CENTER
KANSAS CITY, KS 66160

STEIN, MICHAEL D, MD
PROFESSOR AND CHAIR
DEPARTMENT OF HEALTH LAW, POLICY AND MANAGEMENT
BOSTON UNIVERSITY SCHOOL OF PUBLIC HEALTH
BOSTON, MA 02118

STEINBERG, MARC L, PHD *
ASSOCIATE PROFESSOR
DIVISION OF ADDICTION PSYCHIATRY
ROBERT WOOD JOHNSON MEDICAL SCHOOL
RUTGERS, THE STATE UNIVERSITY OF NEW JERSEY
NEW BRUNSWICK, NJ 08901

TINDLE, HILARY A, MD, MPH
ASSOCIATE PROFESSOR
DIVISION OF INTERNAL MEDICINE AND PUBLIC HEALTH
VANDERBILT UNIVERSITY MEDICAL CENTER
NASHVILLE, TN 37203

TSOH, JANICE Y, PHD
PROFESSOR
DEPARTMENT OF PSYCHIATRY
LANGLEY PORTER PSYCHIATRIC INSTITUTE
UNIVERSITY OF CALIFORNIA SAN FRANCISCO
SAN FRANCISCO, CA 94143

TSUI, JUDITH, MD, MPH *
ASSOCIATE PROFESSOR OF MEDICINE
DIVISION OF GENERAL INTERNAL MEDICINE
UNIVERSITY OF WASHINGTON SCHOOL OF MEDICINE
HARBORVIEW MEDICAL CENTER
SEATTLE, WA 98122

VELASQUEZ, MARY M, PHD
CENTENNIAL PROFESSOR AND DIRECTOR
HEALTH BEHAVIOR RESEARCH
AND TRAINING INSTITUTE
STEVE HICKS SCHOOL OF SOCIAL WORK
UNIVERSITY OF TEXAS AT AUSTIN
AUSTIN, TX 78712

VINCI, CHRISTINE, PHD *
ASSISTANT MEMBER
DEPARTMENT OF HEALTH OUTCOMES AND BEHAVIOR
MOFFITT CANCER CENTER
TAMPA, FL 33612

WINSTANLEY, ERIN L, PHD *
ASSOCIATE PROFESSOR
DEPARTMENT OF BEHAVIORAL MEDICINE AND PSYCHIATRY
WEST VIRGINIA UNIVERSITY, SCHOOL OF MEDICINE
MORGANTOWN, WV 26506

YI, RICHARD, PHD *
PROFESSOR
DEPARTMENT OF PSYCHOLOGY
COFRIN LOGAN CENTER FOR ADDICTION RESEARCH AND
TREATMENT
UNIVERSITY OF KANSAS
LAWRENCE, KS 66045

SCIENTIFIC REVIEW OFFICER

MINTZER, MIRIAM, PHD
SCIENTIFIC REVIEW OFFICER
CENTER FOR SCIENTIFIC REVIEW
NATIONAL INSTITUTES OF HEALTH
BETHESDA, MD 20892

EXTRAMURAL SUPPORT ASSISTANT

FAYEMIWO, TOLU, MS
EXTRAMURAL SUPPORT ASSISTANT
CENTER FOR SCIENTIFIC REVIIEW
NATIONAL INSTITUTES OF HEALTH
BETHESDA, MD 20892

* Temporary Member. For grant applications, temporary members may participate in the entire meeting or may review only selected applications as needed.

Consultants are required to absent themselves from the room during the review of any application if their presence would constitute or appear to constitute a conflict of interest.
